# Bottom-up estimates of reactive nitrogen loss from Chinese wheat production in 2014

**DOI:** 10.1038/s41597-022-01315-4

**Published:** 2022-05-25

**Authors:** Xingshuai Tian, Yulong Yin, Minghao Zhuang, Jiahui Cong, Yiyan Chu, Kai He, Qingsong Zhang, Zhenling Cui

**Affiliations:** College of Resources and Environmental Sciences, China Agricultural University, Beijing, 100193 China

**Keywords:** Agroecology, Element cycles

## Abstract

Excessive use of synthetic nitrogen (N) for Chinese wheat production results in high loss of reactive N loss (Nr; all forms of N except N_2_) into the environment, causing serious environmental issues. Quantifying Nr loss and its spatial variations therein is vital to optimize N management and mitigate loss. However, accurate, high spatial resolution estimations of Nr from wheat production are lacking due to limitations of data generation and estimation methods. Here, we applied the random forest (RF) algorithm to bottom-up N application rate data, obtained through a survey of millions of farmers, to estimate the Nr loss from wheat production in 2014. The results showed that the average total Nr loss was 52.5 kg N ha^−1^ (range: 4.6-157.8 kg N ha^−1^), which accounts for 26.1% of the total N applied. The hotspots for high Nr loss are the same as those high applied N, including northwestern Xinjiang, central-southern Hebei, Shandong, central-northern Jiangsu, and Hubei. Our database could guide regional N management and be used in conjunction with biogeochemical models.

## Abstract (summary)

**Table:** 

Measurement(s)	reactive N loss
Technology Type(s)	random forest model
Sample Characteristic - Environment	cropland
Sample Characteristic - Location	China

## Background & Summary

China is the largest synthetic nitrogen (N) fertilizer producer and consumer in the world, and applied more than 28 Tg N fertilizer to cropland in 2018^1^. Furthermore, China applied 256 kg N ha^−1^ of fertilizer in 2016, which is 3.3 times the global average^2^, while China’s nitrogen use efficiency (NUE) is only 0.25 compared to 0.68 in North America and 0.42 worldwide^3^. A high N input with a low NUE indicates that a considerable amount of N has been lost to environment, mainly in the form of reactive N (Nr; all forms of N except N_2_) including nitric oxide (NO), nitrous oxide (N_2_O), and ammonia (NH_3_) emissions, nitrate (NO_3_^−^) leaching and Nr runoff^4^. This can cause substantial environmental problems, such as soil acidification^5^, air pollution^6^, and eutrophication^7^. the Chinese government has implemented several policies to reduce the environmental risks associated with Nr loss from cropland, such as “zero increase action plan for fertilize use”, and “action plan for organic fertilizer instead of synthetic fertilizer”. These measures are important to optimize N management, improve the NUE, and mitigate Nr loss in China. Understanding Chinese Nr loss at a high-resolution scale is essential to address the variation in N management among crop systems and locations.

Previous studies that aimed to estimate Chinese Nr loss were partially successful^8,9^; however, they had certain limitations that could be addressed. The first limitation concerned the method used for obtaining information on N fertilizer inputs. Fertilizer is distributed to specific locations and crops by regional regulatory bodies based on the total fertilizer input in the entire country or an individual region^10^. Previous studies used information on N fertilizer inputs obtained from regional regularities to estimate Nr loss (top-down information). Although this method can provide rough spatial information for applied N and Nr loss, the application of N is highly location-, and farmer-specific. Consequently, to improve spatial information on Nr loss, an N application rate survey should be used to obtain information from numerous farmers and locations (bottom-up information). The second limitation of previous studies was their focus on NO_3_^−^ leaching, N_2_O and NH_3_ emissions, without consideration of other Nr loss pathways; this led to underestimation of the potential risks of Nr loss^11^. For example, they did not consider NO, one of the most important potential precursors of nitric acid, which leads to acidification and eutrophication^11^. The third limitation of previous studies was that they adopted uniform emission factors (EFs), such as IPCC Tier 1, to estimate the Nr loss of entire countries or regions, rather than considering spatial variation within a country or region^12,13^. Nr loss is location-specific and strongly influenced by local environmental factors. Recent advances have improved spatial estimation of Nr loss by incorporate more environmental factors. For example, Shang *et al*. estimates national cropland-N_2_O emissions by spatially referenced nonlinear model, with spatially variable model parameters depending on environmental factors and crop types^14^. Ying *et al*. applied the random forest (RF) algorithm to estimate the NO_3_^−^ leaching associated with Chinese maize production according to climate and soil variables^15^. These studies indicated that incorporating spatial variation could reduce uncertainties in Nr loss estimations and facilitate management and mitigation decisions. The fourth limitation of previous studies was that they lacked high-resolution Nr emission inventories for specific crops. Such inventories are indispensable for optimal N management.

Wheat is one of the major crops in China, playing a vital role in food security. The regions used for wheat production range from humid regions in the southeast to arid regions in the northwest, and from warm regions in the south to cool regions in the northeast. China accounts for around 20% of the global synthetic N fertilizer consumption for wheat^16^. Considering the substantial spatial variation and excessive N consumption associated with wheat production in China, it represents an excellent target Nr loss estimation methods aiming to overcome the above-mentioned limitations of previous techniques. Our study provides a comprehensive and high-resolution Nr database based on applied synthetic N. First, we developed RF models to predict the EFs of five loss pathways (NO, N_2_O, NH_3_, NO_3_^−^, and Nr runoff) based on a literature review. Second, we use N application rates derived from surveys of 2.23 million farmers to calculate Nr loss. High-resolution data on wheat production distribution in China^17^ are presented in 1 × 1 km grid scale. Our results could help farmers optimize N application within safe boundary and develop mitigation measures against Nr loss in specific locations, and evaluate the environmental effects of Nr loss from Chinese production.

## Methods

### Literature review

**Fig. 1:**
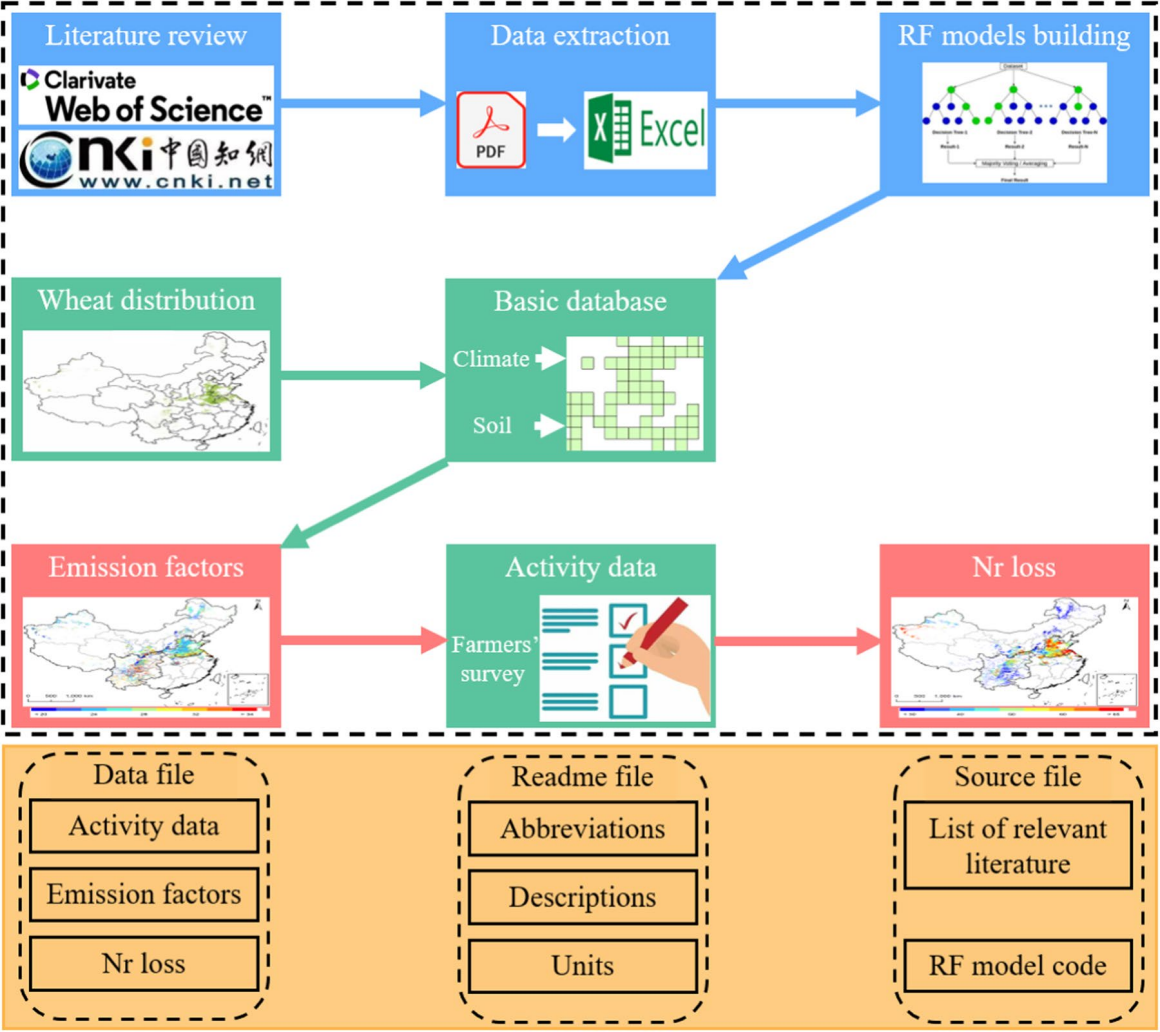
The generate framework of the Nr loss from Chinese wheat system (Nr-Wheat) 1.0 database.

We conducted a comprehensive review of relevant literature published since 1995. Studies were extracted from the China National Knowledge Infrastructure and Web of Science using the following keywords: “N (nitrogen) loss OR NO (nitric oxide) emission OR N_2_O (nitrous oxide) emission OR NH_3_ (ammonia volatilization) emission OR NO_3_^−^ (nitric leaching) OR N (nitrogen) runoff AND wheat AND China”. We excluded the following types of experiment: experiments not covering the entire wheat growing season, experiments conducted in greenhouses or laboratories, experiments without zero-N control, and experiments including manure, controlled release fertilizer, or inhibitors. In total, we extracted 941 observations from 138 articles, consisting of 121 observations of NO emission, 383 of N_2_O emission, 185 of NH_3_ emission, 188 of NO_3_^−^ leaching, and 64 of Nr runoff. We also extracted data on N application rates, and climate and soil variables (Fig. 1). Missing climate data were obtained from China Meteorological Data Network (https://data.cma.cn/), miss values of soil organic carbon (SOC) and total N content were obtained from the National Scientific Fertilizer Network (http://kxsf.soilbd.com/), and missing soil silt, clay, sand content, bulk density, cation exchange capacity (CEC), and pH data were obtained from the Harmonized World Soil Database (HWSD) v. 1.2 (http://www.fao.org/soils-portal/soil-survey/soilmaps-and-databases/harmonized-world-soildatabase-v12/en). Based on this dataset, the EFs of Nr loss pathways were calculated by the following equation:

1 $$E{F}_{i}=\left({E}_{treatment}{\rm{-}}{E}_{control}\right){\rm{/}}N\;applied$$

where *i* = 1–5, represented NO, N_2_O, NH_3_, NO_3_^−^ leaching and Nr runoff, respectively. *E*_*treatment*_ is the loss rate of experimental treatments with applied N fertilizer, *E*_*control*_ is the loss rate of experimental control without applied N fertilizer, and *N applied* is the N application rate corresponding to *E*_*treatment*_. The resulting data was used to develop RF models to predict EFs of the five Nr loss pathways.

### RF models

RF models outperformed empirical models in previous studies^15,18,19^. We employed RF models to predict the EFs of NO, N_2_O, NH_3_, NO_3_^−^ leaching, and Nr runoff. Environmental factors were selected via redundancy analysis^20^. Redundancy analysis, a basic ordination technique for gradients analysis, produces an ordination summarizing the variation in several response variables that can be best explained by a matrix of explanatory variables based on multiple linear regression. We conducted redundancy analysis using Canoco 5 to further analyze the effects of 10 environmental factors, including 4 soil physical factors (bulk density, silt, clay, and sand content), 4 soil chemical factors (pH, SOC, CEC and total N content), and 2 weather factors (total rainfall and mean temperature during the wheat growing period) of different EFs. Ultimately, the dataset of each pathway contained an ensemble of different environmental factors (Table 1).

**Table 1:** Environmental factors were employed to build RF model for each pathway and total explanatory rates.

Loss pathway	Environmental factor	Total explanatory rates (%)
NO	total N content, rainfall, pH, clay, silt, sand	97.8
N_2_O	pH, bulk density, rainfall, SOC, clay, total N content	99.9
NH_3_	clay, rainfall, sand, pH, silt, total N content, temperature, SOC	100.0
NO_3_^-^ leaching	rainfall, total N content, rain, temperature, CEC, pH, sand, clay	99.7
Nr runoff	pH, total N content, temperature, clay, SOC, rainfall	99.9

**Fig. 2:**
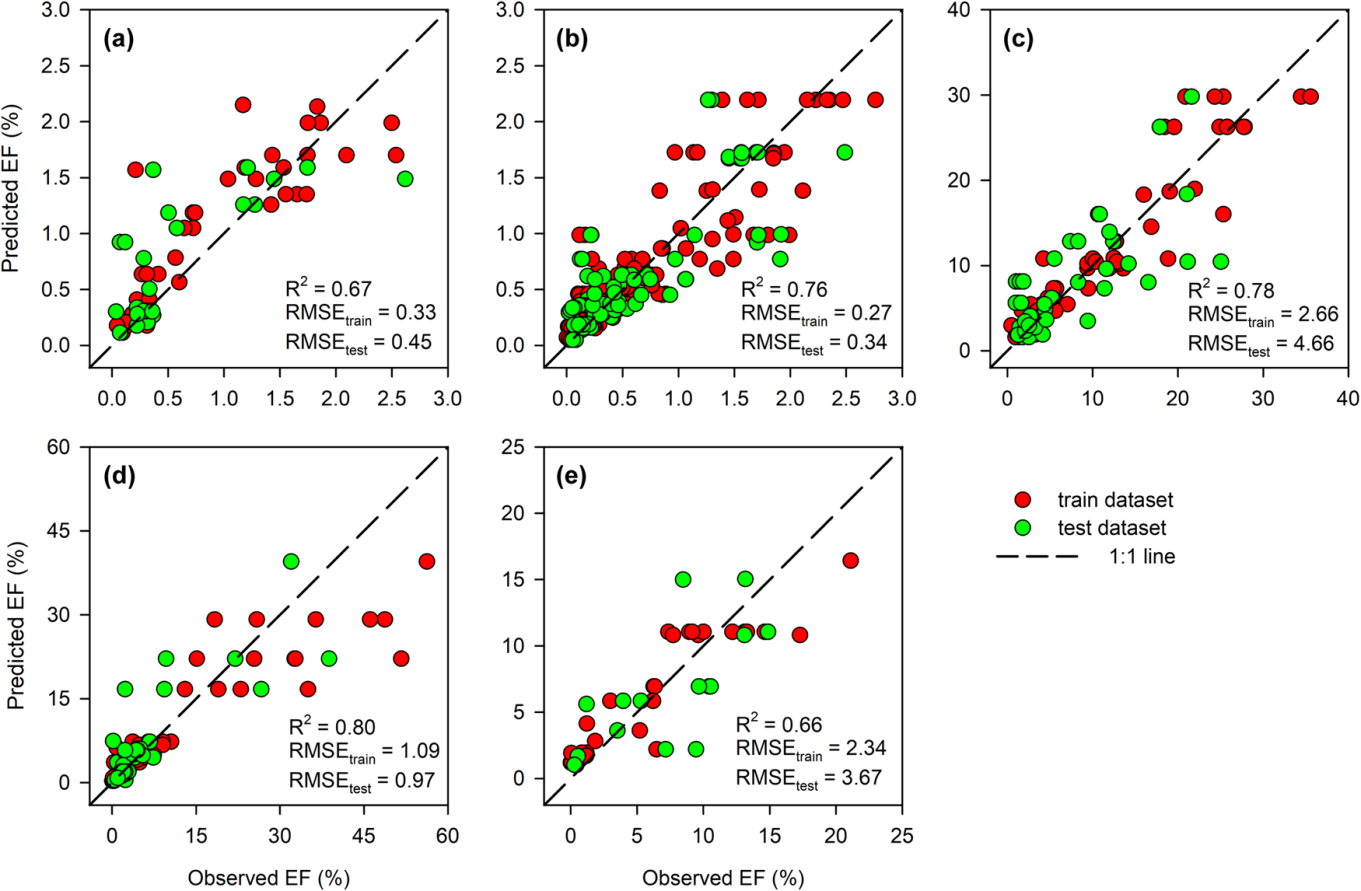
The performance of RF model for each pathway. (**a**) NO, (**b**) N_2_O, (**c**) NH_3_, (**d**) NO_3_^−^ leaching, (**e**) N runoff.

When establishing the RF model, the first step was to select *k* features from a total of *m* (*k* < *m*) in the training dataset, to generate root node d and daughter nodes; the second step was to repeat the first step to generate a forest with *n* decision trees. Lastly, the testing dataset was used to create a final decision tree^21^. We randomly split the dataset, consisting of paired environmental factors and EFs of each Nr loss pathway, into 10 parts of equal size. Among these parts, 7/10 were used to train RF models for different pathways and 3/10 were used to test the performance of the models. We used “*randomForest*” R package (https://www.stat.berkeley.edu/~breiman/RandomForests/) to develop RF models in R software (https://cran.r-project.org/). To reduce random error, we ran each model 500 times and determined the performance based on the average value (Fig. 2).

### Grid database

We categorized Chinese wheat production into four agroecological regions based on climate and soil variables: North China, North China Plain, South China, and Southwest China (Fig. S1)^22^. The grid layer of wheat distribution was derived from ChinaCropArea1 km (10.17632/jbs44b2hrk.2), which provided a 1-km-grid crop-harvest dataset for wheat across China^17^. We selected the grid layer from 2014 and integrated nationwide climate and soil data, and N application rates derived via surveys of farmers, into grid layer (Fig. 1). We obtained climate and soil data from the same sources used for missing data. Climate data are in the form of 10-year averages^23^. The climate and soil data were extracted into each grid and used as input variables for the RF models.

### Predicting EFs and calculating Nr loss

**Fig. 3:**
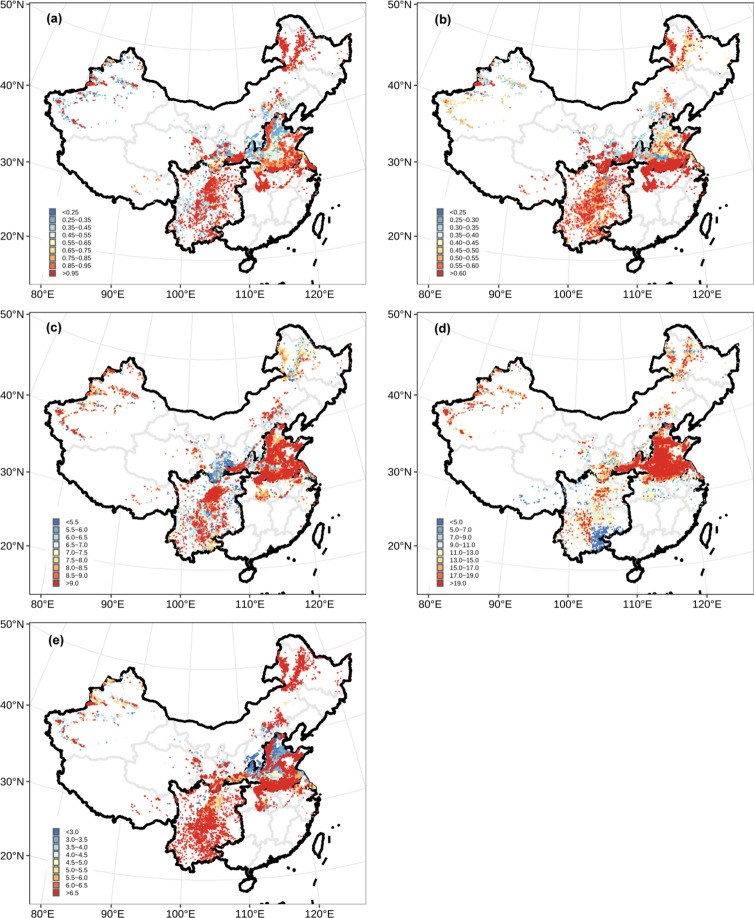
High-resolution (1 × 1 km) patterns of predicted EFs of different Nr loss pathways based on RF models (%). (**a**) NO, (**b**) N_2_O, (**c**) NH_3_, (**d**) NO_3_^−^ leaching, (**e**) Nr runoff.

**Fig. 4:**
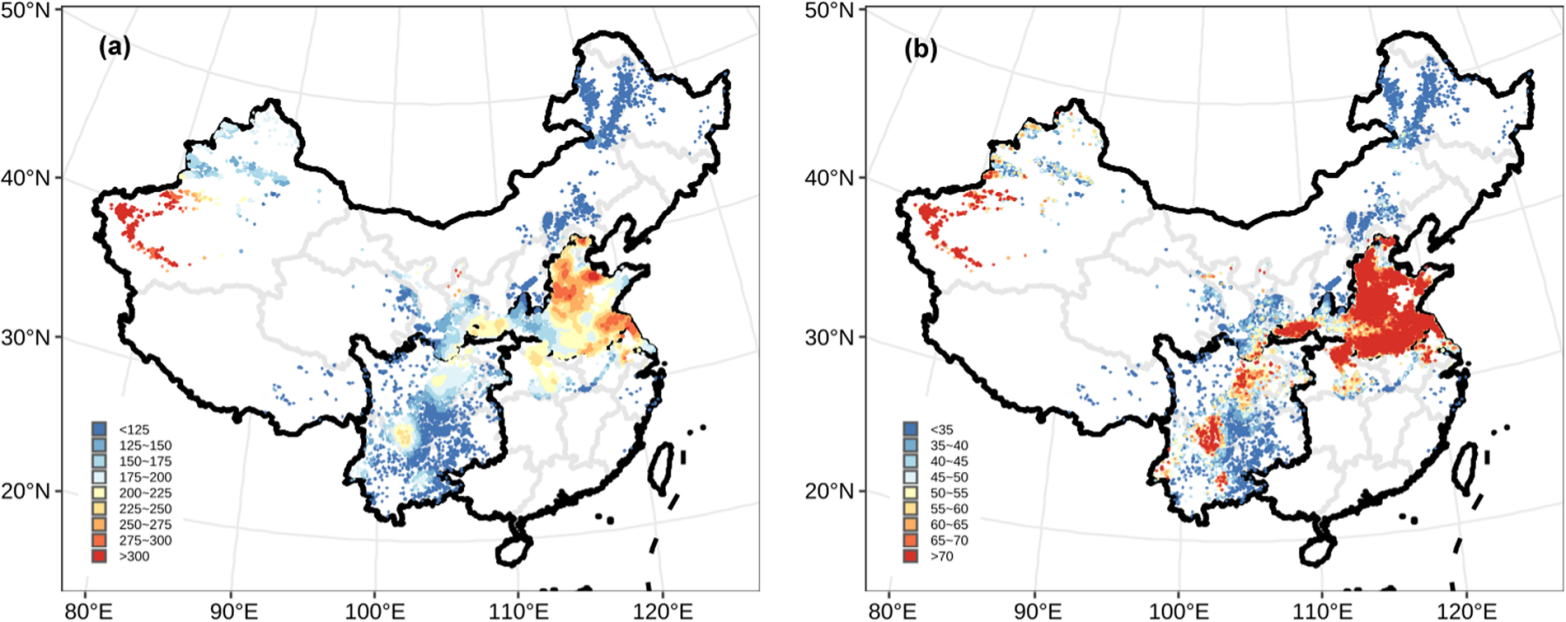
High-resolution (1 × 1 km) patterns of N application rate and total Nr loss. (**a**) N application rate, (**b**) total Nr loss.

**Fig. 5:**
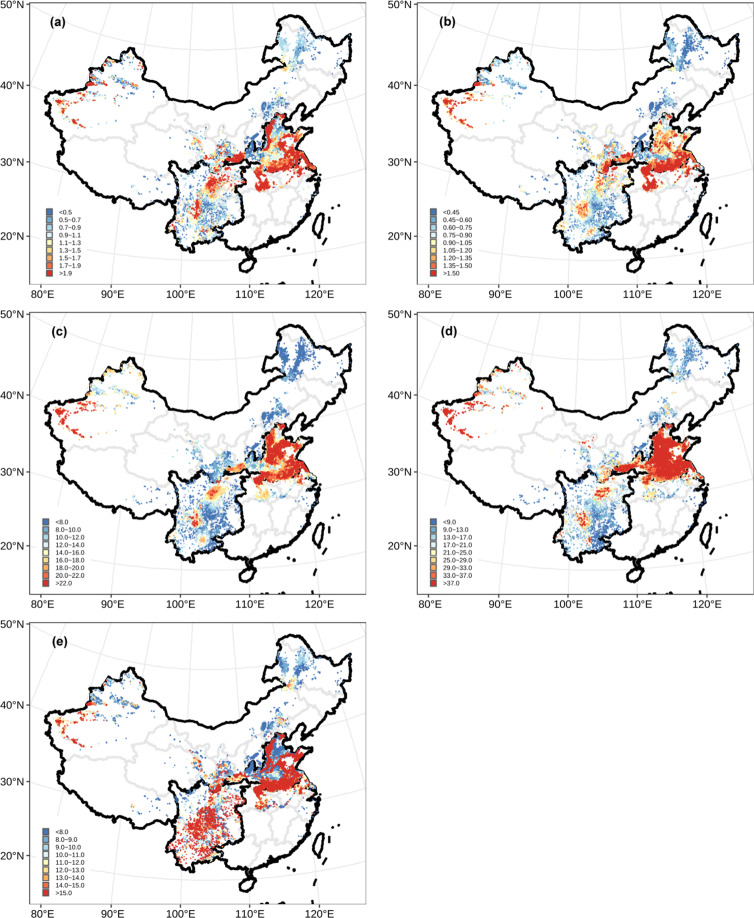
High-resolution (1 × 1 km) patterns of different Nr loss pathways based on RF models (kg N ha^−1^). (**a**) NO, (**b**) N_2_O, (**c**) NH_3_, (**d**) NO_3_^−^ leaching, (**e**) Nr runoff.

The EF of each pathway was predicted by corresponding developed RF model in each grid (Fig. 3). Nr loss was calculated by multiplying predicted EFs by N applied’ using the following equation:

2 $${E}_{ij}=N\;applie{d}_{j}^{{\prime} }\ast \;E{F}_{ij}$$

3 $$total\;Nr\;los{s}_{j}={E}_{1j}+{E}_{2j}+{E}_{3j}+{E}_{4j}+{E}_{5j}+{E}_{6j}$$

where *i* = 1–5, representing NO, N_2_O, NH_3_, NO_3_^−^ leaching and Nr runoff, respectively. And *j* = 1, 2, 3, … represented different grids. *N applied’* was obtained through a nationwide survey of farmers from 2014. For the survey, 3–10 villages were chosen from each county, and 30–120 random farmers were surveyed. In total, 2.23 million farmers from 1,050 counties were surveyed^22^. The N application rates were extracted the average rate was determined for each county, superimposed using Kriging interpolation, and plotted on a map of China. Finally, average rates were extracted into grid layer of Chinese wheat production (Fig. 4a). Total Nr loss (Fig. 4b) was summed from five Nr loss pathways as Eq. (3) (Fig. 5).

### Database structure

The Nr-wheat 1.0 database of Nr loss associated with Chinese wheat production consists of three files (Fig. 1). The ‘data file’ provides N application rates, EFs and Nr loss of five loss pathways (NO, N_2_O, NH_3_, NO_3_^−^, and Nr runoff). The ‘source file’ contains studies from which data were extracted to develop RF models, the code of RF model, and subregions of Chinese wheat production. The ‘readme file’ explains the abbreviations used in the ‘data file’ and ‘source file’, and provides the units of all variables included variables (Fig. 1).

## Data Records

Data records are provided in three files, including ‘source file’, ‘readme file’, and ‘data file’. ‘Source file’ could be found in Supplementary Information, which contained all references used in the database, including 138 relevant papers, the code for the RF model, and four subregions of Chinese wheat cultivation. We divided the relevant papers into 5 subsets based on loss pathways. The ‘readme file’ explained the abbreviations and units. The synthetic N application rates surveyed from farmers, estimated EFs, and Nr loss were integrated into a map and are provided in ‘data file’. The map includes 229,366 1 × 1 km grids, which cover around 94% of wheat crop areas according to official statistics of which approximately 70% are located in the North China Plain. For each pathway, averaged rates and ranges of EFs and Nr loss were summarized (Table 2). The data (‘readme file’ & ‘data file’) can be accessed from National Tibetan Plateau Data Center and processed in ArcGIS, QGIS, R, or Python^24^.

**Table 2:** Averaged values and ranges of EFs and loss for each pathway.

Loss pathway	NO	N_2_O	NH_3_	NO_3_^-^ leaching	Nr runoff	Total
EF (%)	0.5 (0.2–2.2)	0.4 (0.2–1.5)	7.2 (3.1–17.9)	12.2 (1.9–34.0)	5.8 (1.4–23.2)	26.1 (9.0–59.2)
Loss (kg N ha^−1^)	1.0 (0.1–4.6)	0.8 (0.1–3.2)	14.7 (1.9–48.2)	25.0 (1.4–95.3)	11.1 (0.8–55.8)	52.5 (4.6–157.8)

## Technical Validation

Our method and results can be discussed in terms of the (1) data sources, including data extracted from the literature, nationwide climate and soil data, and N application rates derived through surveys of farmers; (2) RF models; and (3) estimated EFs and Nr loss. Regarding (1), all studies from which data were extracted were obtained from authoritative database, including China National Knowledge Infrastructure and Web of Science databases. Each peer-reviewed study was checked by three researchers during the selection process. Nationwide climate and soil data were obtained from Chinese governmental observations and HWSD v1.2, which is widely accepted and used. The N application rates were obtained through surveys of millions of farmers across the entire country; the survey was supported by the Chinese government and many universities, and numerous professional teachers and students from universities were also involved. The data underwent multiple rounds of screening and extensive quality control, and has been published in high-quality international journals^22,25^. Regarding (2), we established RF models for each pathway to predict EFs. All models showed robust performance, with R^2^ values ranging from 0.66–0.80 and low root mean square errors (RMSE) for both training and testing sets (Fig. 2). Regarding (3), the Monte Carlo method was used to estimate the uncertainties of each pathway and total Nr loss; the uncertainties stemmed primarily from predicted EFs and grid-level N application rates. A Monte Carlo simulation was performed to estimate the uncertainty of grid-level N application rates among randomly varying county-level N application rates following Zhou *et al*.^10^, and the results showed that the average coefficient of variation (CV) of grid-level N application rates was 25.8%. The EFs of Nr loss explained more than 60% of the variance in RF models, and the CVs of Nr loss ranged from 20%-34% (Table S1). Assuming normal distributions for grid-level N application rates and EFs, the uncertainties of pathways and total Nr loss were low (Table S2), compared to previous studies^9,26^. Overall, the Nr-Wheat 1.0 database constitutes a robust Nr loss inventory of Chinese wheat production.

## Supplementary information

Supplementary Information for Bottom-up estimates of reactive nitrogen loss from Chinese wheat production in 2014

## Data Availability

All the code used to develop RF model is available in ‘source file’.
