# Supplementary material for: Bottom-up estimates of reactive nitrogen loss from Chinese wheat production in 2014
Source: Sci Data. 2022 May 25;9:233. doi: 10.1038/s41597-022-01315-4 (PMC9133013; doi:10.1038/s41597-022-01315-4)
Supplement: Supplementary file 1 — Supplementary Information for Bottom-up estimates of reactive nitrogen loss from Chinese wheat production in 2014 [file 41597_2022_1315_MOESM1_ESM.pdf]

Supplementary Information for

Bottom-up estimates of reactive nitrogen loss from Chinese wheat production in 2014

Xingshuai Tian, Yulong Yin, Minghao Zhuang, Jiahui Cong, Yiyan Chu, Kai He, Qingsong Zhang, Zhenling Cui*

College of Resources and Environmental Sciences, China Agricultural University, Beijing, 100193, China.

* Corresponding author(s): Zhenling Cui (zhenlingcui@163.com)

The Supplementary Information includes:

Table S1………………………………………………. Page 2

Table S2………………………………………………. Page 3

Figure S1……………………………………………… Page 4

Source file of Nr-Wheat 1.0 database………………… Page 5

Code of RF model…………………………………….. Page 24

Table S1. Coefficient of variation (CV) of each parameter used by Monte Carlo simulation.

Parameter	CV (%)
NO EF	33.0
N_2O EF	24.0
NH_3 EF	22.0
NO_3^- leaching EF	20.0
Nr runoff EF	34.0
N application rates	25.8

Table S2. Uncertainty of each Nr loss and total Nr loss based on Monte Carlo simulation.

Loss pathway	Uncertainty (%)
NO	-72.4 ~ 92.6
N_2O	-60.4 ~ 77.3
NH_3	-59.3 ~ 74.2
NO_3^- leaching	-57.1 ~ 72.0
Nr runoff	-71.8 ~ 97.0
Total Nr loss	-53.0 ~ 59.8

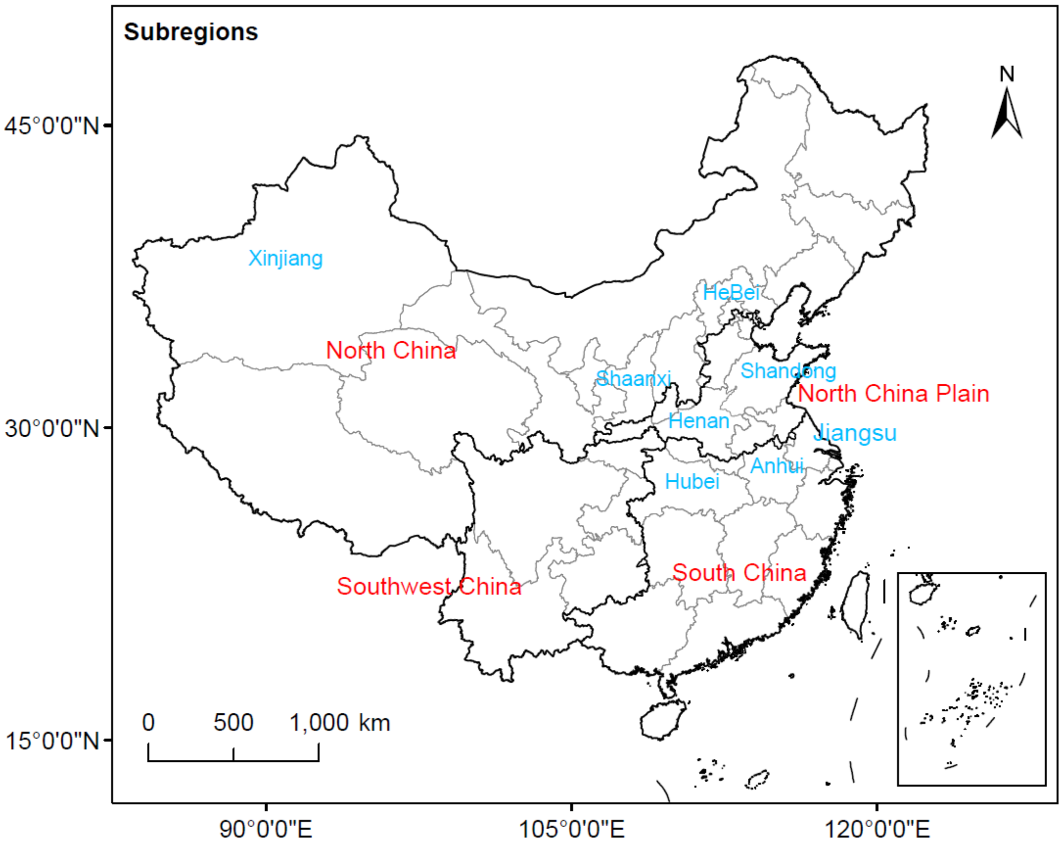

Fig. S1 The subregions of Chinese wheat production and Chinese provincial administrative regions.

Source file: The file contains the information of relevant literatures used for extracting data to develop RF models and code of RF model.

NO emission:

1. Liao, X. et al. Four-year continuous residual effects of biochar application to a sandy loam soil on crop yield and N_2O and NO emissions under maize-wheat rotation. *Agriculture, Ecosystems & Environment*. **302**, 107109 (2020).
2. Lan, T. et al. How are annual CH_4, N_2O, and NO emissions from rice–wheat system affected by nitrogen fertilizer rate and type? *Appl. Soil Ecol.* **150**, 103469 (2020).
3. Tian, D., Zhang, Y., Mu, Y., Liu, J. & He, K. Effect of N fertilizer types on N_2O and NO emissions under drip fertigation from an agricultural field in the North China Plain. *Sci. Total Environ.* **715**, 136903 (2020).
4. Tian, D. et al. Effect of nitrification inhibitors on mitigating N_2O and NO emissions from an agricultural field under drip fertigation in the North China Plain. *Sci. Total Environ.* **598**, 87-96 (2017).
5. Yu, Y. & Zhu, B. Nitric oxide emission and its influencing factors in two paddy-upland rotation systems in Chengdu Plain of Sichuan Basin. *J. Agro-Environ. Sci.* **34**, 1603-1609 (2015). (in Chinese with English abstract)
6. Liu, C., Yao, Z., Wang, K. & Zheng, X. Effects of increasing fertilization rates on nitric oxide emission and nitrogen use efficiency in low carbon calcareous soil. *Agriculture, Ecosystems & Environment*. **203**, 83-92 (2015).
7. Xiang, J., Liu, D., Ding, W., Yuan, J. & Lin, Y. Effects of biochar on nitrous oxide and nitric oxide emissions from paddy field during the wheat growth season. *J. Clean. Prod.* **104**, 52-58 (2015).
8. Zhao, M. et al. Nonlinear response of nitric oxide emissions to a nitrogen application gradient: A case study during the wheat season in a Chinese rice-wheat

rotation system. *Atmos. Environ.* **102**, 200-208 (2015).

9. Liu, L., Cao, Y., Tian, Y., Yin, B. & Zhu, Z. Ammonia volatilization and nitric oxide emission from soil during winter wheat season in Taihu Lake region. *Plant Nutrition and Fertilizer Science.* **19**, 1420-1427 (2013). (in Chinese with English abstract)
10. Lan, T., Han, Y., Roelcke, M., Nieder, R. & Cai, Z. Processes leading to N_2O and NO emissions from two different Chinese soils under different soil moisture contents. *Plant Soil*. **371**, 611-627 (2013).
11. Yan, G. et al. Two-year simultaneous records of N_2O and NO fluxes from a farmed cropland in the northern China plain with a reduced nitrogen addition rate by one-third. *Agriculture, Ecosystems & Environment*. **178**, 39-50 (2013).
12. Cui, F., Yan, G., Zhou, Z., Zheng, X. & Deng, J. Annual emissions of nitrous oxide and nitric oxide from a wheat–maize cropping system on a silt loam calcareous soil in the North China Plain. *Soil Biology and Biochemistry*. **48**, 10-19 (2012).
13. Deng, J. et al. Annual emissions of nitrous oxide and nitric oxide from rice-wheat rotation and vegetable fields: a case study in the Tai-Lake region, China. *Plant Soil*. **360**, 37-53 (2012).
14. Liu, C. et al. Effects of irrigation, fertilization and crop straw management on nitrous oxide and nitric oxide emissions from a wheat–maize rotation field in northern China. *Agriculture, Ecosystems & Environment*. **140**, 226-233 (2011).
15. Zhou, Z. et al. Nitric oxide emissions from rice-wheat rotation fields in eastern China: effect of fertilization, soil water content, and crop residue. *Plant Soil*. **336**, 87-98 (2010).
16. Yao, Z. et al. Effects of tillage during the nonwaterlogged period on nitrous oxide and nitric oxide emissions in typical Chinese rice-wheat rotation ecosystems.

Journal of Geophysical Research. **115**, (2010).

17. Yao, Z. et al. Tillage and crop residue management significantly affects N-trace gas emissions during the non-rice season of a subtropical rice-wheat rotation. *Soil Biology and Biochemistry*. **41**, 2131-2140 (2009).
18. Zheng, X., Huang, Y., Wang, Y. & Wang, M. Seasonal characteristics of nitric oxide emission from a typical Chinese rice–wheat rotation during the non-waterlogged period. *Global Change Biol.* **9**, 219-227 (2003).
19. Zhao, M. et al. Improving agronomic practices to reduce ammonia and nitric oxide emissions from rice-wheat rotation field in Tai Lake Region, China. *Soil.* **47**, 836-841 (2015). (in Chinese with English abstract)

N_2O emission:

1. Hou, M. et al. Effect of organic manure substitution of synthetic nitrogen on crop yield and n2o emission in the winter wheat-summer maize rotation system. *Environ. Sci.* **39**, 321-330 (2018). (in Chinese with English abstract)
2. Htun, Y. M., Tong, Y., Gao, P. & Ju, X. Coupled effects of straw and nitrogen management on N_2O and CH_4 emissions of rainfed agriculture in Northwest China. *Atmos. Environ.* **157**, 156-166 (2017).
3. Huang, T., Yang, H., Huang, C. & Ju, X. Effect of fertilizer N rates and straw management on yield-scaled nitrous oxide emissions in a maize-wheat double cropping system. *Field Crop. Res.* **204**, 1-11 (2017).
4. Niu, Y. et al. Yield-scaled N_2O emissions were effectively reduced by biochar amendment of sandy loam soil under maize - wheat rotation in the North China Plain. *Atmos. Environ.* **170**, (2017).
5. Zhang, A. et al. Contrasting effects of straw and straw–derived biochar application on net global warming potential in the Loess Plateau of China. *Field Crop. Res.*

205, 45-54 (2017).

6. Cheng, G. et al. Comparative analysis on effect of wheat straw and its biochar amendment on net global warming potential under wheat-maize rotation ecosystem in the Guanzhong Plain. *Environmental Science*. **38**, 792-801 (2017). (in Chinese with English abstract)
7. Hao, Y. Effects of long-term fertilization on N_2O emissions and denitrification potential in agricultural soils in Guanzhong Plain. Master thesis, Northwest A & F University, 207. (in Chinese with English abstract)
8. Sun, H., Yan, J., Shi, W. & Zhu, J. Effects of nitrification inhibitor application on wheat grain yield, N_2O Emission and NH_3 volatilization. *Soil*. **49**, 876-881 (2017). (in Chinese with English abstract)
9. Wang, X. et al. Effects of biogas slurry irrigation on CO2 and N2O emission from winter wheat-summer maize rotation farm-land. *J. Agro-Environ. Sci.* **36**, 783-792 (2017). (in Chinese with English abstract)
10. Wei, S. et al. Nitrapyrin-urea application amount with less soil N2O emission and highest profit in summer maize and winter wheat. *Plant Nutrition and Fertilizer Science*. **23**, 231-237 (2017).
11. Xia, L. et al. Integrating agronomic practices to reduce greenhouse gas emissions while increasing the economic return in a rice-based cropping system. *Agr. Ecosyst. Environ.* **231**, 24-33 (2016).
12. Xin et al. Global warming potential and greenhouse gas intensity in rice agriculture driven by high yields and nitrogen use efficiency. *Biogeosciences*, (2016).
13. Yun, L., Wenli, L. & Bo, Z. Effect of fertilization regime on soil N_2O emission from upland field under wheat-maize rotation system., (2016).
14. Zhou, Z., Xin, X., Bi, Z., Lu, L. & Xiong, Z. Soil concentration profiles and

diffusion and emission of nitrous oxide influenced by the application of biochar in a rice-wheat annual rotation system. *Environmental Science & Pollution Research International*. **23**, 7949-7961 (2016).

15. Chen, X. et al. Characteristic variations of N_2O flux in winter wheat field under different tillage methods. *Agricultural Research in The Arid Areas*. **34**, 221-227 (2016). (in Chinese with English abstract)
16. Cui, Z. Research on soil N_2O emission from winter wheat and nitrogen residual effects of wheat on maize season. PhD thesis, Shandong Agricultural University, 2016. (in Chinese with English abstract)
17. Han, J. et al. Contrasting effect of straw return and its biochar on changes in crop yield integrated global warming effects. *Journal of Nanjing Agricultural University*. **39**, 986-995 (2016). (in Chinese with English abstract)
18. Hu, X. Greenhouse gases fluxes of winter wheat-summer maize rotation and mitigation strategies on the North China Plain. PhD thesis, China Agricultural University, 2016. (in Chinese with English abstract)
19. Li, Y. Study on agronomic and environmental effects of combined application of different organic manures with chemical fertilizer. PhD thesis, Chinese Academy of Agricultural Sciences, 2016. (in Chinese with English abstract)
20. Liu, Y., Liu, W. & Zhu, B. Effect of fertilizer regime on soil N_2O emission from upland field under wheat-maize rotation system. *Acta Pedologica Sinica*. **53**, 735-745 (2016). (in Chinese with English abstract)
21. Shan, N. et al. Suitable nitrogen application reducing N_2O emission and improving grain yield in wheat-maize crop rotation system in Beijing suburb. *Transactions of the Chinese Society of Agricultural Engineering*. **32**, 163-170 (2016). (in Chinese with English abstract)

22. Shu, X. et al. Effects of different nitrogen management methods on soil N_2O Emission and crop yield of grain field in North China. *Journal of Agricultural Resources and Environment*. **33**, 340-348 (2016). (in Chinese with English abstract)
23. Tan, Y., Zhuge, Y., Liu, D., Wu, W. & Meng, F. Effect of farmland management on N_2O and CH_4 emission from winter wheat-summer maize rotation system in North China Plain. *Acta Scientiae Circumstantiae*. **36**, 2638-2649 (2016). (in Chinese with English abstract)
24. Huang, P. et al. Coupled water and nitrogen (N) management as a key strategy for the mitigation of gaseous N losses in the Huang-Huai-Hai Plain. *Biol. Fert. Soils*. **51**, 333-342 (2015).
25. Wang, X. et al. Emission characteristics of CH_4 and N_2O fluxes from dryland field under different nitrogen treatments. *Acta entiae Circumstantiae*, (2015).
26. Xiang, J., Liu, D., Ding, W., Yuan, J. & Lin, Y. Effects of biochar on nitrous oxide and nitric oxide emissions from paddy field during the wheat growth season. *J. Clean. Prod.* **104**, 52-58 (2015).
27. Yang, B. et al. Mitigating net global warming potential and greenhouse gas intensities by substituting chemical nitrogen fertilizers with organic fertilization strategies in rice-wheat annual rotation systems in China: A 3-year field experiment. *Ecol. Eng.* **81**, 289-297 (2015).
28. Li, L., Zhou, Z., Pan, X. & Xiong, Z. effects of biochar on N_2O and CH_4 emissions from paddy field under rice-wheat rotation during rice and wheat growing seasons relative to timing of amendment. *Acta Pedologica Sinica*. **52**, 839-848 (2015). (in Chinese with English abstract)
29. Sheng, S. CO_2 and N_2O emission characteristics and impact factors in farmland under biogas slurry irrigation. Master thesis, Chinese Academy of Agricultural

Sciences Dissertation, 2015. (in Chinese with English abstract)

30. Wang, Y., Li, Y., Peng, Z., Wang, C. & Liu, Y. Effects of dicyandiamide combined with nitrogen fertilizer on N_2O emission and economic benefit in winter wheat and summer maize rotation system. *Chinese Journal of Applied Ecology*. **26**, 1999-2006 (2015). (in Chinese with English abstract)
31. Liu, C., Yao, Z., Kai, W. & Zheng, X. Three-year measurements of nitrous oxide emissions from cotton and wheat-maize rotational cropping systems. *Atmos. Environ.* **96**, 201-208 (2014).
32. Qiao, Y. et al. The effect of fertilizer practices on N balance and global warming potential of maize-soybean-wheat rotations in Northeastern China. *Field Crop. Res.* **161**, 98-106 (2014).
33. Xia, L., Wang, S. & Yan, X. Effects of long-term straw incorporation on the net global warming potential and the net economic benefit in a rice-wheat cropping system in China. *Agriculture, Ecosystems & Environment*. **197**, 118-127 (2014).
34. Zhang, Y. F. et al. Effects of controlled release fertilizer on nitrous oxide emission during winter wheat-growing season under no-tillage condition. *Jiangsu Journal of Agricultural sciences*, (2014).
35. Zhou, M. et al. N_2O and CH_4 emissions, and NO_3^- leaching on a crop-yield basis from a subtropical rain-fed wheat–maize rotation in response to different types of nitrogen fertilizer. *Ecosystems*. **17**, 286-301 (2014).
36. Hu, T. Study on GHG emission and mitigation in winter wheat-summer fallow region of south Loess Plateau. Master thesis, Northwest A & F University, 2014. (in Chinese with English abstract)
37. Shan, N. Nitrogen utilization and loss in winter wheat-summer maize rotation system of Beijing suburb. Master thesis, Hebei Agricultural University, 2014. (in

Chinese with English abstract)

38. Hu, X. et al. Greenhouse gas emissions from a wheat-maize double cropping system with different nitrogen fertilization regimes. *Environ. Pollut.* **176**, 198-207 (2013).

39. Huang, T., Gao, B., Christie, P. & Ju, X. Net global warming potential and greenhouse gas intensity in a double-cropping cereal rotation as affected by nitrogen and straw management. *Biogeosciences*. **10**, 7897-7911 (2013).

40. Zhou et al. Nitrous oxide emissions and nitrate leaching from a rain-fed wheat-maize rotation in the Sichuan Basin, China. *Plant & Soil*, (2013).

41. Yan, G. et al. Two-year simultaneous records of N_2O and NO fluxes from a farmed cropland in the northern China plain with a reduced nitrogen addition rate by one-third. *Agriculture, Ecosystems & Environment*. **178**, 39-50 (2013).

42. Liu, D. Optimal fertilization reduced greenhouse gas emissions of wheat-maize cropping system. Master thesis, Shandong Agricultural University, 2013. (in Chinese with English abstract)

43. Zeng, Z., Liu, X., Wang, Y. & Li, D. N_2O emission characteristics of different fertilization treatments in wheat-maize rotation in Hilly Area of Purple Soil. *Journal of Anhui Agricultural Sciences*. **40**, 13777-13780 (2012). (in Chinese with English abstract)

44. Cui, F., Yan, G., Zhou, Z., Zheng, X. & Deng, J. Annual emissions of nitrous oxide and nitric oxide from a wheat–maize cropping system on a silt loam calcareous soil in the North China Plain. *Soil Biology and Biochemistry*. **48**, 10-19 (2012).

45. Ji, Y., Liu, G., Jing, M., Hua, X. & Yagi, K. Effect of controlled-release fertilizer on nitrous oxide emission from a winter wheat field. *Nutr. Cycl. Agroecosys.* **94**, 111-122 (2012).

46. Liu, C., Wang, K. & Zheng, X. Responses of N2O and CH4 fluxes to fertilizer nitrogen addition rates in an irrigated wheat-maize cropping system in northern China. *Biogeoences*. **9**, 839-850 (2012).

47. Ji, Y. et al. Effect of controlled-release fertilizer (CRF) on nitrous oxide emission during the wheat growing period. *Acta Pedologica Sinica*. **49**, 526-534 (2012). (in Chinese with English abstract)

48. Ma, Y. et al. Effects of N fertilization rates on the NH_3 volatilization and N_2O emissions from the wheat-maize rotation system in North China Plain. Ecology and Environment Sciences. **21**, 225-230 (2012). (in Chinese with English abstract)

49. Huang, J., Zhang, Y., Liu, H. & Wang, B. CO_2 and N_2O emissions from red soil during wheat and corn growing seasons under different patterns of long-term fertilization. *Journal of Ecology and Rural Environmen*t. **27**, 7-13 (2011). (in Chinese with English abstract)

50. Zhai, L., Liu, H., Zhang, J., Huang, J. & Wang, B. Long-Term Application of Organic Manure and Mineral Fertilizer on N_2O and CO_2 Emissions in a Red Soil from Cultivated Maize-Wheat Rotation in China. *Agricultural Sciences in China*. **10**, 1748-1757 (2011).

51. Shi, P. Effects of long-term application of fertilizer on wheat yield and emission of the CO_2 and N_2O from soil in loess plateau. Master thesis, Northwest A & F University, 2011. (in Chinese with English abstract)

52. Jiang, J., Hu, Z., Sun, W. & Huang, Y. Nitrous oxide emissions from Chinese cropland fertilized with a range of slow-release nitrogen compounds. *Agriculture, Ecosystems & Environment*. **135**, 216-225 (2010).

53. Liu, S., Qin, Y., Zou, J. & Liu, Q. Effects of water regime during rice-growing season on annual direct N_2O emission in a paddy rice–winter wheat rotation system

in southeast China. *Sci. Total Environ.* **408**, 906-913 (2010).

54. Wei, X. R. et al. Nitrous oxide emission from highland winter wheat field after long-term fertilization. *Biogeosciences*. **7**, 3301-3310 (2010).

55. Yao, Z. et al. Effects of tillage during the nonwaterlogged period on nitrous oxide and nitric oxide emissions in typical Chinese rice-wheat rotation ecosystems. *Journal of Geophysical Research: Biogeosciences*, (2010).

56. Xue, X. Effect of fertilization on nitrate leaching and greenhouse gases emission in the typical dry-farming area. PhD thesis, University of Chinese Academy of Sciences, 2010. (in Chinese with English abstract)

57. Yao, Z. et al. Tillage and crop residue management significantly affects N-trace gas emissions during the non-rice season of a subtropical rice-wheat rotation. *Soil Biol. Biochem.* **41**, 2131-2140 (2009).

58. Zhao, X. et al. Nitrogen fate and environmental consequence in paddy soil under rice-wheat rotation in the Taihu lake region, China. *Plant & Soil*. **319**, 225-234 (2009).

59. Wang, X. et al. Effect of optimized nitrogen application on ammonia volatilization from soil in winter wheat-summer corn rotation system in Northern China. *Plant Nutrition and Fertilizer Science*. **15**, 344-351 (2009). (in Chinese with English abstract)

60. Chen, S., Huang, Y. & Zou, J. Relationship between nitrous oxide emission and winter wheat production. *Biol. Fert. Soils*. **44**, 985-989 (2008).

61. Chen, L. Research on nitrogen recycle and its effect on environment in dry-land of Loess Plateau. Master thesis, Northwest A & F University, 2007. (in Chinese with English abstract)

62. Li, X. Fate of fertilizer nitrogen and gaseous N loss in winter wheat-summer maize

rotation system in North China Plain. Master thesis, Hebei Agricultural University, 2007. (in Chinese with English abstract)

63. Zou, J., Huang, Y., Lu, Y., Zheng, X. & Wang, Y. Direct emission factor for NO from rice–winter wheat rotation systems in southeast China. *Atmos. Environ.* **39**, 4755-4765 (2005).
64. Pan, Z., Wu, W., Liu, G. & Gao, X. Effect of straw return and nitrogen fertilizer application on the N_2O emission of soil. *Soil and Fertilizers*, 6-8 (2004). (in Chinese with English abstract)
65. Liang, D. Nitrous oxide losses of nitrogen fertilizer and influential factors on loess soil. PhD thesis, Northwest A & F University, 2003. (in Chinese with English abstract)

NH_3 emission:

1. Xu, C. Characteristics of greenhouse gas emissions and nitrogen losses under long-term nitrogen fertilization and straw incorporation in the North China Plain. PhD thesis, China Agricultural University, 2018. (in Chinese with English abstract)
2. Dong, Y., Wu, Z., Li, B., Xu, X. & Xiong, Z. Effects of biochar reapplication on ammonia volatilization and nitrogen use efficiency during wheat season in a rice-wheat annual rotation system. *Plant Nutrition and Fertilizer Science.* **23**, 1258-1267 (2017). (in Chinese with English abstract)
3. Jing, J. Ammonia volatilization of winter wheat canopy under different nitrogen rates. Master thesis, Northwest A & F University, 2017. (in Chinese with English abstract)
4. Zheng, F. Effects of long-term application of chemical fertilizer and organic manure on nitrogen flow and water use efficiency in winter wheat field. Master thesis, Shandong Agricultural University, 2017. (in Chinese with English abstract)

5. Sun, H., Yan, J., Shi, W. & Zhu, J. Effects of nitrification inhibitor application on wheat grain yield, N_2O emission and NH_3 volatilization. *Soil.* **49**, 876-881 (2017). (in Chinese with English abstract)
6. Zheng, F., Dong, S., Liu, P., Zhang, J. & Zhao, B. Effects of combined application of manure and chemical fertilizers on ammonia volatilization loss and yield of winter wheat. *Plant Nutrition and Fertilizer Science.* **23**, 567-577 (2017). (in Chinese with English abstract)
7. Zhang, B. et al. Effects of chlorine-containing nitrogen fertilizer on ammonia volatilization and yields under rice-wheat rotation system in Taihu Lake region. *Plant Nutrition and Fertilizer Science.* **23**, 557-566 (2017). (in Chinese with English abstract)
8. Zhang, W. Study on effect of thiourea amine on crop yield and ammonia volatilization in a rice-wheat rotation system. Master thesis, Sichuan Agricultural University, 2016. (in Chinese with English abstract)
9. Gu, L. et al. Lysimeter study of nitrogen losses and nitrogen use efficiency of Northern Chinese wheat. *Field Crop. Res.* **188**, 82-95 (2016).
10. Guo, X. et al. Effects of controlled-release fertilizer dosage and depth on wheat yield and nitrogen utilization efficiency in reclamation land. *Chinese Journal of Soil Science.* **47**, 928-934 (2016). (in Chinese with English abstract)
11. Wang, H. et al. Suppression of ammonia volatilization from rice-wheat rotation fields amended with controlled-release urea and urea. *Agron. J.* **108**, 1214-1224 (2016).
12. Wang, X., Zhou, W., Liang, G., Pei, X. & Li, K. The fate of 15N-labelled urea in an alkaline calcareous soil under different N application rates and N splits. *Nutr. Cycl. Agroecosys.* **106**, 311-324 (2016).

13. Huang, P. et al. Coupled water and nitrogen (N) management as a key strategy for the mitigation of gaseous N losses in the Huang-Huai-Hai Plain. *Biol. Fert. Soils*. **51**, 333-342 (2015).

14. Li, Q. et al. Effect of a new urease inhibitor on ammonia volatilization and nitrogen utilization in wheat in north and northwest China. *Field Crop. Res.* **175**, 96-105 (2015).

15. Shan, N. et al. Ammonia volatilization from wheat soil under different nitrogen rates. *J. Agro-Environ. Sci.* **33**, 1858-1865 (2014). (in Chinese with English abstract)

16. Shan, N. Nitrogen utilization and loss in winter wheat-summer maize rotation system of Beijing suburb. Master thesis, Hebei Agricultural University, 2014. (in Chinese with English abstract)

17. Zhai, X. et al. Ammonia volatilization loss in Huang Huai winter wheat cultivation areas under irrigated and rainfed conditions. *Plant Nutrition and Fertilizer Science.* **19**, 54-64 (2013). (in Chinese with English abstract)

18. Ma, Y. et al. Effects of N fertilization rates on the NH_3 volatilization and N_2O emissions from the wheat-maize rotation system in North China Plain. *Ecology and Environment Sciences.* **21**, 225-230 (2012). (in Chinese with English abstract)

19. Wu, G. Effect of nitrogen fertilizer management in wheat growth season on nitrogen utilization and residual effect in winter wheat and summer maize cropping system. PhD thesis, Shandong Agricultural University, 2012. (in Chinese with English abstract)

20. Lu, Y. & Song, F. Effects of different coated controlled-release urea on soil ammonia volatilization in farmland. *Acta Ecologica Sinica.* **31**, 148-155 (2011). (in Chinese with English abstract)

21. Ji, Y. et al. Ampact of different nitrogen application on nitrogen movement and gaseous loss of winter wheat fields. *Journal of Soil and Water Conservation.* **24**, 113-118 (2010). (in Chinese with English abstract)
22. Xia, W. et al. Effect of optimized nitrogen application on ammonia volatilization from paddy field under wheat-rice rotation system. *Plant Nutrition and Fertilizer Science.* **16**, 6-13 (2010). (in Chinese with English abstract)
23. Li, F. The effect of long-tern fertilization on wheat yield and the ecological environment in the dry-land of Loess Plateau. Master thesis, Northwest A & F University, 2009. (in Chinese with English abstract)
24. Wang, X. et al. Effect of optimized nitrogen application on ammonia volatilization from soil in winter wheat-summer corn rotation system in Northern China. *Plant Nutrition and Fertilizer Science.* **15**, 344-351 (2009). (in Chinese with English abstract)
25. Huang, J., Ge, G. & Fan, X. Nitrogen use efficiency and ammonia volatilization from urea applied to wheat on a paddy field in Taihu Lake region. Journal of Anhui Agricultural University. **36**, 677-682 (2009). (in Chinese with English abstract)
26. Pang, F. Effects of combined application of organic and inorganic fertilizers on soil ammonia volatilization and nitrate accumulation in winter wheat yield. Master thesis, Chinese Academy of Agricultural Sciences, 2008. (in Chinese with English abstract)
27. Wang, X., Zhu, J., Gao, R., Yasukazu, H. & Feng, K. Nitrogen cycling and losses under rice-wheat rotations with coated urea and urea in the Taihu Lake region. *Pedosphere*. **17**, 62-69 (2007).
28. Li, X. Fate of fertilizer nitrogen and gaseous N loss in winter wheat-summer maize rotation in North China Plain. Master thesis, Hebei Agricultural University, 2007.

(in Chinese with English abstract)

29. Chen, L. Research on nitrogen recycle and its effect on environment in dry-land of Loess Plateau. Master thesis, Northwest A & F University, 2007. (in Chinese with English abstract)
30. Wang, D., Yu, Z., Yu, W., Shi, Y. & Zhou, Z. Effects of nitrogen application level on soil nitrate accumulation and ammonia volatilization in high-yielding wheat field. *Chinese Journal of Applied Ecology*, 1593-1598 (2006). (in Chinese with English abstract)
31. Liu, X., Ju, X., Zhang, F., Pan, J. & Christie, P. Nitrogen dynamics and budgets in a winter wheat–maize cropping system in the North China Plain. *Field Crop. Res.* **83**, 111-124 (2003).

NO_3^- leaching:

1. Kuang, F. Fate of N fertilizer and N balance in different cropping systems in purple soil areas of Upper Reaches of Yangtze River. PhD thesis, China Agricultural University, 2016. (in Chinese with English abstract)
2. Ding, Y. Characteristics of N leaching and apparent N budget in cultivated lands under a winter wheat-summer maize rotation system in Guanzhong Plain. Master thesis, Northwest A & F University, 2015. (in Chinese with English abstract)
3. Cao, Y., Tian, Y., Yin, B. & Zhu, Z. Improving agronomic practices to reduce nitrate leaching from the rice–wheat rotation system. *Agriculture, Ecosystems & Environment*. **195**, 61-67 (2014).
4. Shan, N. Nitrogen utilization and loss in winter wheat-summer maize rotation system of Beijing suburb. Master thesis, Hebei Agricultural University, 2014. (in Chinese with English abstract)
5. Zhou, M. et al. N_2O and CH_4 Emissions, and NO_3^- Leaching on a crop-yield basis

from a subtropical rain-fed wheat–maize rotation in response to different types of nitrogen fertilizer. *Ecosystems*. **17**, 286-301 (2014).

6. Tan, D. et al. An in situ study of inorganic nitrogen flow under different fertilization treatments on a wheat–maize rotation system surrounding Nansi Lake, China. *Agr. Water Manage.* **123**, 45-54 (2013).
7. Yang, X. N budget of a cultivated land in wheat-maize rotation district on the Guanzhong Plain of Shaanxi province. Master thesis, Northwest A & F University, 2013. (in Chinese with English abstract)
8. Ni, X., Liang, X., Tian. G., Huang, J. & Shi, Y. Effects of different nitrogen fertilizer rates on nitrate leaching characteristics and wheat yield in paddy field in dry period. *Acta Agriculturae Zhejiangensis.* **24**, 670-675 (2012). (in Chinese with English abstract)
9. Yu, Y., Xue, L. & Yang, L. Nitrogen use efficiency and loss from runoff and leaching in wheat season with rice-wheat rotation system under different nitrogen management methods in Taihu Lake Region, China. *J. Agro-Environ. Sci.* **30**, 2475-2482 (2011). (in Chinese with English abstract)
10. Huang, M. et al. Leaching losses of nitrate nitrogen and dissolved organic nitrogen from a yearly two crops system, wheat-maize, under monsoon situations. *Nutr. Cycl. Agroecosys.* **91**, 77-89 (2011).
11. Yin, X. Characteristics of nitrogen fertilizer utilization and nitrate-N leaching in the Guanzhong Area. Master thesis, Northwest A & F University, 2010. (in Chinese with English abstract)
12. Zhao, X. et al. Nitrogen fate and environmental consequence in paddy soil under rice-wheat rotation in the Taihu lake region, China. *Plant Soil.* **319**, 225-234 (2009).

13. Zhang, J., Wang, D. & Wang, C. On nutrient leaching amount of rice-wheat rotation field with monolith lysimeter in Taihu Lake Area. *Soil*, 591-595 (2008). (in Chinese with English abstract)
14. Chen, L. Research on nitrogen recycle and its effect on environment in dry-land of Loess Plateau. Master thesis, Northwest A & F University, 2007. (in Chinese with English abstract)
15. Fang, Q. et al. Soil nitrate accumulation, leaching and crop nitrogen use as influenced by fertilization and irrigation in an intensive wheat–maize double cropping system in the North China Plain. *Plant Soil*. **284**, 335-350 (2006).
16. Zhao, R. The sustainability evaluation of the optimized management of water and nitrogen resources for winter wheat-summer maize rotation system. PhD thesis, China Agricultural University, 2006. (in Chinese with English abstract)
17. Mao, G., Lu, M., Huang, M. & Liu, M. Nitrogen loss in rice-wheat cropping farmland and its control measures. *Acta Agriculturae Shanghai*, 86-92 (2006). (in Chinese with English abstract)
18. Wang, X., Gao, R., Zhu, J., Bao, C. & Feng, K. Nitrogen loss via runoff and leaching from soil as a function of types of urea applied in wheat season. *Rural Eco-Environment*, 24-29 (2005). (in Chinese with English abstract)
19. Yi, S. Study on nitrogen leaching loss and utilization in rice-wheat/oil rotation System. Master thesis, Southwest University, 2005. (in Chinese with English abstract)
20. Yi, S., Shi, X., Wen, M., Li, X. & Huo, Q. Nitrogen transference and leaching loss in growth period of wheat in purple soil. *Journal of Soil and Water Conservation*, 46-49 (2004). (in Chinese with English abstract)
21. Liu, X., Ju, X., Zhang, F., Pan, J. & Christie, P. Nitrogen dynamics and budgets in

a winter wheat–maize cropping system in the North China Plain. *Field Crop. Res.* **83**, 111-124 (2003).

22. Wang, D., Lin, J. & Xia, L. Characteristics of nitrogen leaching of rice-wheat rotation field in Taihu Lake area. *Chinese Journal of Eco-Agriculture*, 26-28 (2001). (in Chinese with English abstract)
23. Yu, G., Mao, Z., Shi, X. & Liu, H. Study of N migration and leaching loss in purple soils. *Journal of Southwest Agricultural University*, 22-26 (1999). (in Chinese with English abstract)

Nr runoff:

1. Wang, X. et al. Comparative of two fertilization modes on crop yields and nitrogen/phosphorus runoff losses under rice-wheat rotation. *Journal of Soil and Water Conservation.* **34**, 20-27 (2020). (in Chinese with English abstract)
2. Lou, Y., Zhu, G., Zhao, H. & Gao, Z. Study on characteristics of nitrogen and phosphorus loss in runoff of wheat-rice rotation in Anhui Eastern Area. *Journal of Anhui Agricultural Sciences.* **48**, 78-80 (2020). (in Chinese with English abstract)
3. Qin, X. et al. Effects of chemical fertilizer reduction combined with biogas fertilizer on crop yield of wheat–maize rotation and soil nitrogen loss in North China Plain. *J. Agro-Environ. Sci.* **39**, 1558-1567 (2020). (in Chinese with English abstract)
4. Cao, Y. et al. Reducing N losses through surface runoff from rice-wheat rotation by improving fertilizer management. *Environ. Sci. Pollut. R.* **24**, 4841-4850 (2017).
5. Chen, Q. et al. Characteristics of nitrogen and phosphorus runoff losses in organic and conventional rice-wheat rotation farm-land in Taihu Lake Region. *J. Agro-Environ. Sci.* **35**, 1550-1558 (2016). (in Chinese with English abstract)
6. Tan, D. et al. Effects of fertilization controlling nitrogen and phosphorus loss from

farmland under wheat-maize rotation in Nansi Lake Region. *Acta Pedologica Sinica.* **52**, 128-137 (2015). (in Chinese with English abstract)

7. Xue, L., Yu, Y. & Yang, L. Maintaining yields and reducing nitrogen loss in rice-wheat rotation system in Taihu Lake region with proper fertilizer management. *Environ. Res. Lett.* **9**, 115010 (2014).
8. Zhao, X. et al. Nitrogen runoff dominates water nitrogen pollution from rice-wheat rotation in the Taihu Lake region of China. *Agriculture, Ecosystems & Environment*. **156**, 1-11 (2012).
9. Wang, G. et al. Study of nitrogen and phosphorus runoff in wheat-rice rotation farmland in Chao Lake Basin. *Journal of Soil and Water Conservation.* **24**, 6-10 (2010). (in Chinese with English abstract)
10. Tian, Y., Yin, B., Yang, L., Yin, S. & Zhu, Z. Nitrogen Runoff and Leaching Losses during Rice-Wheat Rotations in Taihu Lake Region, China. *Pedosphere*. **17**, 445-456 (2007).
11. Zhu, X., Sheng, H., Xia, X. & Wang, Y. Nitrogen loss with runoff and drainage from farmland under wheat-rice rotation. *Journal of Ecology and Rural Environment.* 38-41 (2006). (in Chinese with English abstract)

Code of RF model

```
# loading packages
library(rio)
library(tidyverse)
library(randomForest)
# loading data
# data1: data of NO; data2:data of N2O; data3: data of NH3; data4: data of NO3-
leaching; data5: data of Nr runoff
data<-import("data3.xlsx",sheet="sheetname")
head(data)
head(data)
set.seed(123456)
i=1
repeat{
train_data<-sample(nrow(data),7/10*nrow(data))
train<-data[train_data,]
test<-data[-train_data,]
fit.forest<-randomForest(EF~.,train,importance=TRUE,mtry=3,ntrees=1000)
fit.forest
train_pred<-predict(fit.forest,newdata=train)
test_pred<-predict(fit.forest,newdata=test)
i=i+1
if (i > 500){
    break
    }
```

```
}
# Prediction by RF model (fit.forest)
grid_database<-import("grid_database.xlsx")
prediction<-predict(fit.forest,newdata=grid_database)
export(predition,"prediction.xlsx")
# Other processes were conducted in MS Excel and Arcgis
```
